# Discerning Primary and Secondary Factors Responsible for Clinical Fatigue in Multisystem Diseases

**DOI:** 10.3390/biology3030606

**Published:** 2014-09-22

**Authors:** David Maughan, Michael Toth

**Affiliations:** Department of Molecular Physiology & Biophysics, University of Vermont, Burlington, VT 05405, USA; E-Mail: mtoth@uvm.edu

**Keywords:** muscular fatigue, multi-system diseases, muscle deconditioning, chronic fatigue syndrome, multiple sclerosis, cancer, heart failure

## Abstract

Fatigue is a common symptom of numerous acute and chronic diseases, including myalgic encephalomyelitis/chronic fatigue syndrome, multiple sclerosis, heart failure, cancer, and many others. In these multi-system diseases the physiological determinants of enhanced fatigue encompass a combination of metabolic, neurological, and myofibrillar adaptations. Previous research studies have focused on adaptations specific to skeletal muscle and their role in fatigue. However, most have neglected the contribution of physical inactivity in assessing disease syndromes, which, through deconditioning, likely contributes to symptomatic fatigue. In this commentary, we briefly review disease-related muscle phenotypes in the context of whether they relate to the primary disease or whether they develop secondary to reduced physical activity. Knowledge of the etiology of the skeletal muscle adaptations in these conditions and their contribution to fatigue symptoms is important for understanding the utility of exercise rehabilitation as an intervention to alleviate the physiological precipitants of fatigue.

## 1. Introduction

Fatigue is a symptom often associated with multi-system diseases, including myalgic encephalomyelitis/chronic fatigue syndrome, cancer, multiple sclerosis, heart failure, obstructive pulmonary disease, lupus, and Lyme disease. The fatigue symptoms are generally attributed to some combination of metabolic, neurological, and myofibrillar adaptations. Numerous pathophysiological adaptations associated with these conditions impact skeletal muscle function in ways that may promote muscle fatigue, but it is not clear to what extent many of the adaptations are simply secondary to muscle disuse [1,2,3]. Additionally, it is unclear what role that muscle fatigue may play in the symptomatic fatigue in these disorders. In the discussion below, we start with a brief review of pathological syndromes reported for four diseases that have fatigue as a defining symptom. We focus specifically on skeletal muscle fatigue and the cellular and molecular adaptations that may contribute to this fatigue, with a brief discussion of the confounding effects of uncomplicated disuse on skeletal muscle. We conclude with a commentary that emphasizes the need, in future research, to differentiate between the effects of the disease *versus* those of disuse on skeletal muscle adaptations.

Before we discuss specific examples of diseases that are characterized by fatigue, we need to define fatigue. This is a difficult task because, in the context of clinical populations, there are multiple adaptations that occur in neural, metabolic, muscle and other systems that occur and combine to form the complex clinical presentation of patient-reported, subjective fatigue. Thus, for the purposes of this review, we define “clinical fatigue” as a subjective sensation of physical tiredness or exhaustion that is disproportionate to the level of activity. This is the classical definition of clinical or symptomatic fatigue and is of clear importance to the treating physician, as this sensation of fatigue can have diagnostic significance or provoke alterations in treatment course. Unfortunately, patient-reported fatigue stems not only from fatiguing stimuli in the affected tissues and organs, but from a complex interaction between the central and peripheral neural mechanisms that perceive fatiguing stimuli. Additionally, the relative contribution of generated *versus* perceived fatiguing stimuli likely differs from disease to disease, as well as patient to patient. Thus, there is no easy way to dissociate the generation and perception of fatiguing stimuli in the context of a real-world clinical condition.

Nonetheless, in many clinical conditions, physiologic adaptations in fatigued muscles undoubtedly contribute to the etiology of clinical fatigue [4]. In this review we focus on skeletal muscle adaptations that might reasonably be expected to contribute to clinical fatigue, either via limiting physiological capacity, promoting muscle fatigue or both. Thus we chose to define “muscle fatigue” as a drop in physical work capacity (*i.e.*, reduced force or power production) in response to contractile activity [5]. We highlight three main physiological parameters that contribute most prominently to a phenotype that predisposes to diminished physiological capacity: muscle size, oxidative capacity, and contractile function. Diminution of any one of these individually or in combination will likely require any given activity to be performed at a greater percentage of maximal capacity and, in turn, will generate more fatiguing stimuli. Our focus on these three elements is not meant to exclude other potentially important modulators (e.g., neural activation, excitation-contraction coupling, *etc.*), but, primarily, because of those factors intrinsic to skeletal muscle, these are the most well-studied attributes that determine the physiological capacity of skeletal muscle in humans and, in turn, generation of stimuli that would contribute to both muscle and clinical fatigue.

## 2. Skeletal Muscle Adaptations in Chronic Multi-System Diseases

### 2.1. Myalgic Encephalomyelitis/Chronic Fatigue Syndrome (ME/CFS)

We begin our discussion with a condition for which the hallmark-defining symptom is fatigue. Myalgic encephalomyelitis [6], often referred to as Chronic Fatigue Syndrome in the United States [7], is a devastating neuroimmune disease [8,9] displaying global disruption of the nervous, immune and endocrine systems [6]. Approximately 0.4%–1% of the adult US population has ME/CFS [10], although the percentage may be far higher considering the lack of wide-spread recognition of the disease in the general population and by the medical community. Symptoms include marked physical and cognitive fatigue, unrefreshing sleep, and a prolonged recovery period in response to even modest physical or mental activity. Muscle pain and fatigue are common symptoms, even at rest. Patients often develop fibromyalgia, a related neuroimmune disorder distinguished by chronic widespread pain and allodynia (a heightened and painful response to pressure) [11].

Abnormalities are evident within the immune [12] and central nervous [13] systems that likely stem from defective oxidative and nitrosative pathways and a lower antioxidant status [14,15]. Mitochondrial function is depressed, with the severity of the disease correlating with lower oxidative phosphorylation, nucleotide transport, and ATP levels in blood neutrophils [16,17]. There is some evidence that compromised metabolic function extends to skeletal muscles [18] and other major organs [16]. In what may be a compensatory response, anaerobic metabolism is up-regulated via enhanced glycolysis [16,17]. The regulation may be structurally based in supramolecular complexes of glycolytic and glycogenolytic enzymes [19]. Cytoplasmic compartmentation and the formation of enzyme complexes probably boosts ATP production and, with further regulatory enhancement, may help alleviate the depressed aerobic metabolism evident in ME/CFS. However, any benefits of shifting from oxidative to glycolytic pathways may be offset, during periods of increased physical activity, by excess production of fatigue-producing metabolic by-products (phosphate and metabolic acids) [20].

Metabolic defects may also be reflected in abnormalities in blood flow regulation and mitochondrial function, some of which may be linked to altered endothelial nitric oxide (NO) [21] and hydrogen sulfide (H_2_S) [22] metabolism. NO relaxes the smooth muscles that surround arterioles and arteries, increasing the flow of blood when required. In ME/CFS patients, reduced NO production by endothelial cells [21] may increase the constriction of arterioles and arteries, whereas a postulated deregulation of H_2_S [22] may lead to an inhibition of cytochrome-c oxidase and thus a reduction in mitochondrial production of ATP. A reduced blood flow or mitochondrial ATP production in critical organs, including the skeletal muscles, brain, and brain stem, could elicit a variety of somatosensory symptoms of ME/CFS, including a diminished ability to perform physical activity [23].

Skeletal muscle fatigue, the topic of interest here, likely contributes to post-exertional fatigue in ME/CFS. A small shift from fatigue-resistant, oxidative type I fibers towards oxidative, type II fibers occurs in some patients, with little or no attendant atrophy [24]. Nuclear magnetic resonance [25,26] and electromyography [27] reveal pathological features that are consistent with defective ion channel or receptor function [28,29,30]. Skeletal muscle mitochondrial function may also be blunted, as it is in blood neutrophils [16]. Oxidative stress [14,31] or autoantibodies [32] directed against mitochondrial proteins, plasma membrane proteins, or metabolic enzymes may play a role in the ME/CFS pathophysiology—all of which would lead to diminished physical activity. In addition, oxygen delivery to the patient’s skeletal muscles is impaired [33], contributing to the metabolic insufficiency observed in the musculature of ME/CFS patients [34]

In evaluating ME/CFS-related muscle fatigue, it is unclear to what extent aging and deconditioning contributes to the disease phenotype. Incorporation of these variables (particularly the former) into reported studies has generally been ignored. Research focusing on this issue is sparse, although one recent report shows diminished function of ventilatory muscles during exercise in ME/CFS patients that appears to be attributable to deconditioning [35].

Further complicating the picture, it is unclear to what extent alterations in somatosensory input and higher brain function contribute to muscle fatigue [13,36]. Cognitive function, which drives the sensation of fatigue, clearly has a role in contributing to a debilitating condition, but the mechanisms are uncertain. Some studies report impairment of cognitive function after exercise [37,38] but others do not [39,40]. However, when activity and cognitive function are closely and continuously monitored, physical and psychological symptoms appear to be dissociated [41].

### 2.2. Multiple Sclerosis (MS) (Aka, Encephalomyelitis Disseminate)

MS has remarkable phenomenological and neuroimmune overlaps with ME/CFS [9], although MS is distinguished by the anatomical hallmark of a progressive destruction of the myelin coating of axons. The prevalence of MS is about half that of ME/CFS. Both MS and ME/CFS patients experience severe levels of disabling fatigue and a worsening of symptoms following exercise. Autonomic dysfunction, diminished cardiac responses and a relapsing-remitting or progressive course, with infections and psychosocial stress, play a part in driving the fatigue symptoms. Both MS and ME/CFS exhibit evidence of autoimmunity and activated immunoinflammatory, oxidative and nitrosative pathways [42,43]. As in ME/CFS, lower levels of ATP, decreased phosphocreatine synthesis and impaired oxidative phosphorylation are linked to mitochondrial dysfunction [44,45,46]. NMR studies show similarities in CNS pathology, including decreased cerebral blood flow, atrophy, gray matter reduction, white matter hyperintensities, increased cerebral lactate and choline signaling, and lowered acetyl-aspirate levels [47,48,49] (and references therein).

Physical and mental activity usually increase fatigue, although the response is generally not as severe as with ME/CFS [9]. Nevertheless, many MS patients experience rapid exhaustion and exercise intolerance which can lead to profound disability [50]. Fatigability of striated muscle of a kind that is not related to CNS activity is a frequent manifestation of MS [51]. In one study, for example, exercise produced declines in tetanic force, phosphocreatine and intracellular pH that were greater in patients than in controls, without a concomitant change in compound muscle action potential amplitude, further supporting the notion that clinical fatigue is in part due to alterations in the intracellular contractile and ionic milieu of MS patients [52]. The skeletal muscles in MS patients are also significantly smaller than those in healthy individuals and, as in ME/CFS [16], rely more on anaerobic respiration than in healthy controls [8,9]. Several studies have revealed abnormalities reflecting defects in maximal voluntary contraction [53,54] that indicate altered muscle contractile function, as well as impaired oxidative metabolism and atrophy. In more advanced stages of the disease, pathological sequelae of MS clearly alter skeletal muscle structure and function. However, one notable pathological feature of MS that differs from ME/CFS (and that undoubtedly impacts upon the skeletal muscle phenotype) is impaired central neural activation of muscles secondary to neurodegeneration [55]. This feature of the disease is more evident in advanced stages of neurodegeneration where motor function is grossly impaired. With *de facto* denervation, skeletal muscle disuse ensues with a variety of accompanying adaptations. In this context, one must pay careful attention to subject selection to clearly delineate factors related to the disease and neural insufficiency.

As in ME/CFS, in evaluating MS-related muscle fatigue it is unclear to what extent aging and deconditioning contributes to the disease phenotype. To date, MS-related studies appear to have generally ignored these variables, although a possible link between an increased fatigability, impaired metabolic response [51] and reduced physical activity [52] has been noted in MS patients [56]. Questions of aging and disuse are generally discussed in relation to morphological, biochemical, and physiological changes observed in pre-clinical (animal) and human studies that address these variables, as, for example, in examining the effects of immobilization [52] on muscle performance. Nevertheless, at least some portion of the muscle adaptations to MS and its treatment may be explained by disuse. Aging, upon which the time course of disease and disuse is superimposed, is clearly another contributor to fatigue, but the relative extent to which each variable contributes to the clinical and physiological phenotypes is, at best, speculative. The challenge of discerning the extent to which muscle-specific phenotypes relate to all three contributors is addressed at the end of this review (Section 3).

### 2.3. Cancers

Fatigue is one of the most prevalent and distressing side effects of cancer and its treatment [57,58] and is estimated to affect 60%–90% of patients [59]. Cancer-related fatigue can lead to dose reductions or cessation of cancer treatments and is a predictor of chemotherapy toxicity and survival [60,61,62,63,64]. Despite the profoundly detrimental effects of cancer-related fatigue, our current understanding of the factors contributing to decrements in physiological capacity that lead to this condition is incomplete, with most data coming from animal models. Clinically, cancer-related fatigue encompasses both the effects of cancer and its treatment since, in human patients, the two are inextricably linked.

Approximately half of cancer patients experience weight loss during the disease, with muscle atrophy being an essential contributor [65]. The loss of muscle size extends to the cellular level [66], and studies in animal models [67] and patients [68] further suggest that gross muscle fiber atrophy may also be accompanied by a selective loss of the contractile protein myosin. However, more recent studies in animal models and humans have suggested that loss of myosin is likely a methodological artifact, and that the loss extends to other myofilament proteins [66,69]. Regardless, the loss of muscle size reduces the amount of contractile tissue available to perform physical work, which manifests functionally as both muscle weakness and reduced aerobic fitness. Parenthetically, recent studies suggest that cancer is not associated with a shift in muscle fiber type distribution as reflected in altered MHC isoform expression [66], arguing against a role for alterations in fiber type admixture contributing to declines in muscle functional capacity.

Cancer patients are generally assumed to experience muscle weakness during the disease, but the data are surprisingly sparse. Assessments of whole muscle strength and size in cancer patients [70,71] and animal models [72,73,74] indicate a reduction in muscle force production, although it is unclear to what extent observed contractile dysfunction (*i.e.*, reduced intrinsic function per unit muscle size) is directly linked to the primary lesion, cancer. In a population of mostly lung cancer patients, we recently found 25% lower knee extensor isometric torque after statistical adjustment for muscle mass [66], without loss of myofilament protein or alterations in fiber type. Moreover, reduced knee extensor strength was strongly related to decreased power output during a walking endurance test [66], underscoring the potential relevance of muscle weakness to fatigue in daily activities. Further evidence of intrinsic muscle contractile dysfunction was provided in these same studies at the cellular and molecular levels, as we found that cancer was associated with reduced tension in fast-twitch myosin heavy chain (MHC) IIA fibers, which was explained by a reduction in the number of strongly-bound myosin-actin cross-bridges. Additionally, we found a profound reduction of myosin-actin cross-bridge kinetic parameters in slow-twitch, MHC I fibers, as evidenced by an increase in myosin attachment time. We would predict that shortening velocity would be reduced in MHC I fibers because it is inversely related to attachment time. Collectively, these reductions in velocity in MHC I fibers and force in MHC IIA fibers, in turn, would reduce power output in both fiber types and, presumably, the whole muscle.

There is also evidence for reduced aerobic capacity in skeletal muscle in cancer patients. Recent experiments from our laboratory have shown that cancer is associated with a 50% reduction in mitochondrial fractional area and a 37% reduction in average mitochondria size compared to healthy controls [66]. Moreover, lower mitochondrial content was associated with single fiber contractile dysfunction and tends to be associated with reduced walking performance in cancer patients [66], suggesting a potential contribution of mitochondrial abnormalities to muscle weakness and fatigability [75]. Further compounding reductions in mitochondrial content, cancer [76,77] and chemotherapeutic agents [78] have been shown to impair mitochondrial function in pre-clinical models. Taken together, mitochondrial loss and dysfunction represent adaptations that may contribute to cancer-related fatigue.

The extent to which these adaptations in muscle size, contractility and mitochondrial content/function relate to cancer and its treatment, or occur secondary to the physical inactivity that accompanies the disease, are unclear. Physical activity is reduced in cancer patients following diagnosis and during treatment [79] (and references therein), but there is a surprising lack of studies that have evaluated skeletal muscle adaptations and activity levels longitudinally in cancer patients to discern the potential contribution of muscle disuse to cancer-related fatigue. Data from exercise training studies, although not equivalent to simply preventing declines in habitual physical activity, suggest that reductions in muscle function during treatment in breast cancer patients can be diminished [80], arguing that at least some portion of the muscle adaptations to cancer and its treatment may be explained by disuse. Defining this contribution of disuse to skeletal muscle adaptations that may contribute to the broader syndrome of cancer-related fatigue has implications for the utility of exercise training in countering this debilitating symptom of the disease and its treatment. In particular, as randomized controlled studies move exercise rehabilitation towards standard of care in cancer patients [81], understanding the muscle adaptations that occur and their contribution to muscular fatigue and, in turn, clinical fatigue will permit the optimization of exercise interventions to remediate this important and debilitating symptom.

### 2.4. Cardiovascular Disorders and Heart Failure

Exercise intolerance is a common problem in a variety of cardiovascular disorders, although its impact is generally not as severe as it is in ME/CFS, MS, and cancer (albeit, absent acute disease exacerbation). Exercise intolerance—defined subjectively as dyspnea and fatigue upon exertion and clinically as reduced peak oxygen uptake (peak VO_2_) during an incremental exercise test—is the cardinal symptom of chronic heart failure (HF). HF represents the final common pathway for most chronic cardiovascular disease. While reduced VO_2_ was originally attributed to cardiac insufficiency, work over the last three decades has led to an appreciation of non-cardiac, or peripheral, adaptations to the HF syndrome that contribute to exercise intolerance [82]. In particular, these peripheral adaptations reflect clear myopathic changes in skeletal muscle [83], encompassing alterations in structure and function.

Perhaps the most well-described adaptation is a reduction in skeletal muscle size, which has been observed both at the whole muscle [84] and cellular [85] levels. The specific protein metabolic adaptations that promote these reductions in muscle size are not clearly known [86], but they undoubtedly contribute to exercise intolerance in HF patients. Strong correlations have been found between muscle mass and peak VO_2_ [87]. We recognize that a physiological measurement such as aerobic fitness does not explain completely what makes an individual “fatigued” in response to a given activity, but it is clear that any reduction in aerobic capacity will mean that a given activity will be performed at a greater percentage of their maximal work capacity and, as such, may generate more fatigue-inducing metabolic by-products and be perceived as more fatiguing.

Another well-characterized component of the skeletal muscle myopathy of HF is diminished oxidative capacity. This is signaled at the whole body level as a reduction in aerobic fitness, as described above, but is also observed at the cellular level, as a shift from slow-twitch, oxidative muscle fibers towards fast-twitch, more glycolytic fibers [88], with a concomitant shift in fatigue-producing metabolic by-products, as in ME and MS (see above). At the organellar level, studies have also shown reduced mitochondrial function [20,84] and content [89] in HF patients. Thus, both the overall oxidative character of the muscle, owing to gross fiber type changes, and the intrinsic oxidative capacity of existing muscle fibers, appears to be diminished with heart failure. Since skeletal muscle oxidative capacity is an important determinant of whole body oxygen consumption, these adaptations may partially explain the diminished aerobic fitness level mentioned above.

One final adaptation in HF that may contribute to fatigue is diminished muscle contractile function. This has been well-characterized at the whole muscle level [87]. Although at least some proportion of muscle weakness in HF patients is likely explained by atrophy, there are persistent deficits that cannot be explained by muscle loss [87]. Moreover, this weakness is not accompanied by deficiencies in neural activation [90]. In fact, recent studies have observed reduced single muscle fiber contractile function from HF patients secondary to alterations in the amount and function of myofilament proteins [91,92]. Thus, there appears to be a unique effect of HF on the intrinsic function of the more basic elements of the contractile machinery.

The question of the contribution of disease-related deconditioning/disuse in the muscle phenotype observed in HF patients is perhaps more well-developed than in any other disease state [93]. This is because several laboratories have undertaken studies in which patients with HF were matched to controls with similar habitual physical activity levels or degree of cardiorespiratory deconditioning, but no cardiac pathology. Results from these studies have demonstrated that the shift in muscle fibers towards a more fast-twitch phenotype, mitochondrial loss and diminished mitochondrial oxidative function in HF patients are no longer apparent [92,94,95,96], implying that these muscle phenotypes likely occur secondary to disease-related muscle disuse rather than to HF per se, albeit there are deficits that persist and may be fundamentally related to features of the disease process, such as muscle contractile dysfunction [92,97]. Nonetheless, these studies highlight the modulating effects of muscle disuse/deconditioning on muscle biology in HF patients. If these disuse-related muscle adaptations contribute to exercise intolerance, exercise rehabilitation may be a suitable intervention to counteract this hallmark of disease symptomology.

## 3. Disease and Muscle Deconditioning

Common triggers of multi-system, fatigue-producing diseases, such as those discussed above, include viruses, bacteria, physical and mental stressors. While the triggers may differ, the final symptoms are often alike, resulting in a broad class of related diseases that cover a wide spectrum of etiologies. Common pathways leading to comparable post-translational modifications of proteins and enzymes, oxidative stress, inflammation, and hypoperfusion produce phenotypical outcomes, which, as noted in this commentary, can be remarkably similar—including skeletal muscle structural and functional adaptations that might be expected to contribute to muscle fatigue and the broader syndrome of clinical fatigue. Overlaying the direct impact of the disease on skeletal muscle function is the confounding variable of muscle disuse, which, of its own accord, produces secondary changes in muscle structure and function. While muscle fatigue is generally listed as part of the symptom profile of these multi-system diseases, the contribution of muscle deconditioning to clinical fatigue is rarely considered or accounted for in research studies. The lingering question is: to what extent is symptomatic muscle fatigue due to the primary disease and to what extent is it secondary to reduced physical activity? This is an important question because the answer helps define the therapeutic approach to muscle fatigue.

The challenge ahead for designing therapies to ameliorate clinical fatigue in multi-system diseases will be a multi-step process. First, we must identify what role skeletal muscle structural and functional adaptations in each condition contribute to muscle fatigue and, in turn, how these adaptations, either directly through their tendency to increase the production of fatiguing stimuli, or indirectly through the contribution of muscle fatigue, impact clinical fatigue, and second, to develop strategies to differentiate fatigue related to chronic muscle disuse from fatigue related to the primary disease process. Finally, although not discussed in-depth in this review, because the perception of fatiguing stimuli plays an essential role in the complex syndrome of clinical fatigue the mechanisms underlying the sensory recognition of fatiguing stimuli must be clearly understood. In this regard, new approaches will be helpful, such as one recently reported by Pollak *et al.* [98], who discovered that two types of sensory neurons detect ATP, protons, and lactate (metabolic substrates and products in muscle contraction): one type detects low concentrations of metabolites and is associated with the perception of exercise-induced fatigue, while the other type detects higher levels of metabolites and is associated with the perception of pain. Combinations of these metabolites found in resting muscles evoked no sensations, whereas combinations found in muscle during moderate endurance exercise produced significant fatigue sensations, which were even stronger during vigorous exercise. The highest levels of metabolites (as found in ischemic exercise) produced significantly more muscle pain, but no greater sensation of fatigue. Clearly methodological approaches such as these would be helpful in identifying modalities and neural pathways involved in fatigue perception in multi-system diseases. Correlating results of such studies with functional assays at multiple levels should, by providing a clearer understanding of clinical fatigue, motivate a new wave of therapeutic approaches.

**Figure 1:**
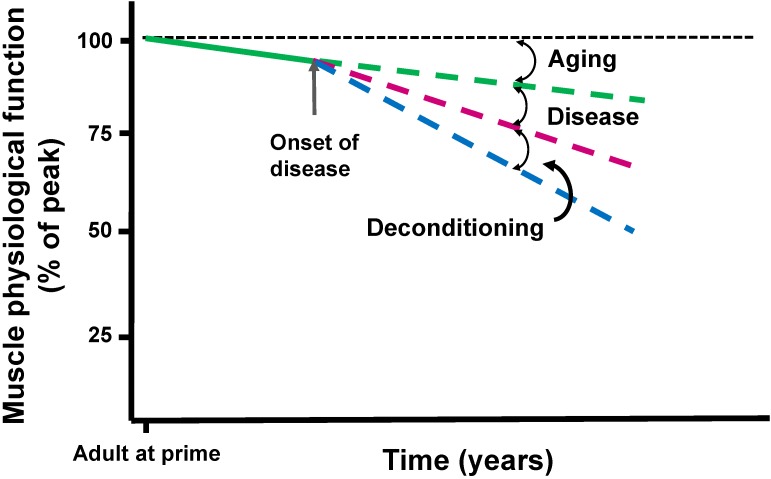
Physiological function (represented here as a composite index of muscle size, strength, and oxidative capacity, relative to maximum) declines with age (green hatched line), to which declines from disease (magenta hatched line) and muscle deconditioning (blue hatched line) produce a cumulative phenotype. The rates of decline in this figure are hypothetical and do not represent actual data. The *primary disease effect* is simply what the disease would present in the absence of muscle deconditioning. Exercise rehabilitation may partially or fully correct the effect of muscle deconditioning, except in the case of ME/CFS, where standard therapeutic approaches involving exercise may in fact exacerbate the condition. In general, it is not clear whether exercise therapy can also blunt or reverse aspects of the age-related decline in physiological function. While the sensation of fatigue tracks muscle physiological function, the magnitude of the sensation (relative to the maximum experienced) may be proportionally greater than the proportionate loss of physiological function.

Figure 1 summarizes the challenge that researchers face in discerning the extent to which disease-related muscle phenotypes related to the primary disease *versus* muscle disuse—and the extent to which rehabilitation exercise therapy may correct or reverse the progressive development of muscle fatigue. The hypothetical time lines depict the primary effect of the disease itself (magenta hatched line) and the secondary effect of deconditioning (blue hatched line) on muscle physiological function, superimposed on the inevitable decline of function due to aging (green hatched line). The cumulative fatigue phenotype is the sum of all three. Exercise rehabilitation, which essentially counteracts the muscle disuse/deconditioning that accompanies many diseases, may be able to effectively remediate that specific component of the cumulative fatigue phenotype (difference between blue and red line). While this general approach undoubtedly cannot alleviate all of the symptomology of the condition, it may provide some symptomatic relief and allow patients to retain a higher level of functionality.

An exception to the general utility of exercise rehabilitation is the one multi-system disease in which chronic fatigue is the hallmark symptom: ME/CFS. Even *graded* exercise therapy [99] is known to exacerbate ME/CFS by placing too much stress on the compromised systems, leading to a worsening of symptoms which may be injurious [100]. What is the recommended approach to easing muscle fatigue in ME/CFS? Proper nutrition combined with dietary supplements as needed, restorative sleep, and carefully pacing one’s activities so as not to overtax the body [36].

In all cases, reduced muscle physiological function resulting from the muscle adaptations described above would lower the physiological capacity for work and, in turn, make any given activity appear more difficult. The sensation of fatigue likely parallels reductions in physiological function, although the *sensation* of fatigue may be, in terms of the proportional effects of disease and muscle disuse, far greater than the physiological effects. In conclusion, what one sees with chronic disease is a slide into less functionality, with diminished muscle size, strength, and oxidative capacity. As a bulwark against this slide, exercise becomes particularly important for maintaining/improving muscle size, contractility and oxidative capacity, all of which help to ameliorate fatigue by reducing the amount of fatiguing stimuli. Where exercise is tolerated, it is beneficial. The only possible exception is ME/CFS, where, as noted above, restorative rest and careful pacing of one’s activities are foremost among the currently recommended therapeutic approaches.
